# Endoplasmic Reticulum (ER) Stress and Unfolded Protein Response (UPR) in Mammalian Oocyte Maturation and Preimplantation Embryo Development

**DOI:** 10.3390/ijms20020409

**Published:** 2019-01-18

**Authors:** Tao Lin, Jae Eun Lee, Jung Won Kang, Hyeon Yeong Shin, Ju Bin Lee, Dong Il Jin

**Affiliations:** Division of Animal & Dairy Science, Chungnam National University, Daejeon 34134, Korea; ailuomansi@gmail.com (T.L.); lje9090@hanmail.net (J.E.L.); rinstein@naver.con (J.W.K.); sin-hy@cnu.ac.kr (H.Y.S.); dlwnqlssla@naver.com (J.B.L.)

**Keywords:** endoplasmic reticulum stress, unfolded protein response, oocytes, embryos, apoptosis

## Abstract

Mammalian oocytes and early embryos derived from in vitro production are highly susceptible to a variety of cellular stresses. During oocyte maturation and preimplantation embryo development, functional proteins must be folded properly in the endoplasmic reticulum (ER) to maintain oocyte and embryo development. However, some adverse factors negatively impact ER functions and protein synthesis, resulting in the activation of ER stress and unfolded protein response (UPR) signaling pathways. ER stress and UPR signaling have been identified in mammalian oocytes and embryos produced in vitro, suggesting that modulation of ER stress and UPR signaling play very important roles in oocyte maturation and the development of preimplantation embryos. In this review, we briefly describe the current state of knowledge regarding ER stress, UPR signaling pathways, and their roles and mechanisms in mammalian (excluding human) oocyte maturation and preimplantation embryo development.

## 1. Introduction

In eukaryotic cells, the endoplasmic reticulum (ER) is the major intracellular organelle responsible for protein synthesis [1,2]. The accumulation of misfolded or unfolded proteins in the ER lumen can disrupt ER homeostasis and activate ER stress [3,4]. The unfolded protein response (UPR) is an adaptive mechanism of the ER; its activation allows cells to respond to ER stress conditions [5,6]. Although the precise molecular mechanisms of the UPR remain poorly described in mammalian embryo development [4], it is generally accepted that the UPR is regulated by three major sensors: Double-stranded RNA-activated protein kinase-like ER kinase (PERK), activating transcription factor 6 (ATF6) and inositol-requiring enzyme 1 (IRE1) [7]. As a pro-survival response, the UPR works to alleviate the accumulation of misfolded proteins and restore ER function [8]. However, in cases where ER stress becomes prolonged or too severe for UPR-based mitigation, apoptosis is triggered.

New protein synthesis due to the translation of maternal mRNA is extremely important for oocyte maturation and embryo development [9,10]. During oocyte maturation and preimplantation embryo development, the ER acts as a major site for the biosynthesis of proteins, lipids and secretory proteins, and thus plays a key role in meeting the oocyte’s increased demand for new proteins. These functional proteins must be folded properly in the ER to maintain appropriate oocyte maturation and embryo development. Thus, regulation of ER stress/homeostasis is likely to be an important mechanism during these processes [9].

Developing gametes and embryos may encounter various types of exogenous stress in an in vitro culture system [11,12]. It is becoming more and more apparent that some of these adverse factors negatively impact ER functions and protein synthesis, resulting in the activation of ER stress and the UPR signaling pathways [11]. Indeed, ER stress and UPR signaling appear to play critical roles during oocyte meiotic resumption and preimplantation embryo development. Inhibition of UPR signaling by ER stress inhibitors has been shown to improve oocyte maturation and early embryo development in pigs, mice, bovines, etc. [4,13,14,15,16], suggesting that ER stress is detrimental to mammalian oocyte maturation and preimplantation embryo development. Thus, understanding the mechanistic relationships between ER stress and in vitro development could help the improvement of the maturation of oocytes and early development of embryos.

This review examines what currently is known regarding the involvement, potential impacts and mechanisms of ER stress and UPR signaling in mammalian (excluding human) oocyte maturation and preimplantation embryonic development.

## 2. Endoplasmic Reticulum Stress and Unfolded Protein Response Signaling Pathways

### 2.1. Endoplasmic Reticulum Stress and the Unfolded Protein Response

**Figure 1:**
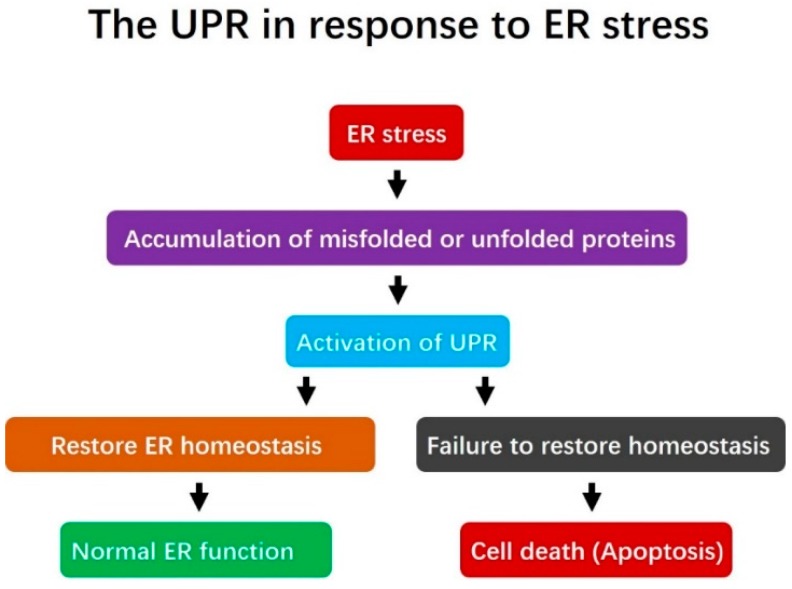
The role of the unfolded protein response (UPR) in addressing Endoplasmic Reticulum (ER) stress. Under ER stress, misfolded or unfolded proteins accumulate in the ER. This triggers activation of the UPR, which is a pro-survival response designed to alleviate the accumulation of misfolded proteins, restore ER homeostasis and re-establish normal ER function. However, if the UPR fails to restore ER homeostasis, cell death (apoptosis) is induced.

The ER is a multifunctional essential organelle found in eukaryotic cells. It is a major site for the synthesis of transmembrane proteins and lipids, and is involved in maintaining intracellular calcium homeostasis [1,2,17]. The ER quality control (ERQC) system is in charge of identifying properly folded proteins, which are channeled for transport to the Golgi complex, versus misfolded proteins, which are retained in the ER to undergo correct folding or be targeted for degradation by the ER-associated degradation (ERAD) machinery [18]. Correct protein folding is one of the most important steps during protein synthesis, and the accumulation of misfolded proteins in the ER lumen disturbs ER functions and leads to ER stress. Activation of ER stress can trigger the UPR, which is a cascade of adaptive pathways that seek to maintain cellular homeostasis and normal ER function. However, if ER stress is severe or prolonged, the UPR is incapable of re-establishing ER homeostasis and normal function, and apoptosis may occur (Figure 1).

### 2.2. The Unfolded Protein Response Signaling Pathway

**Figure 2:**
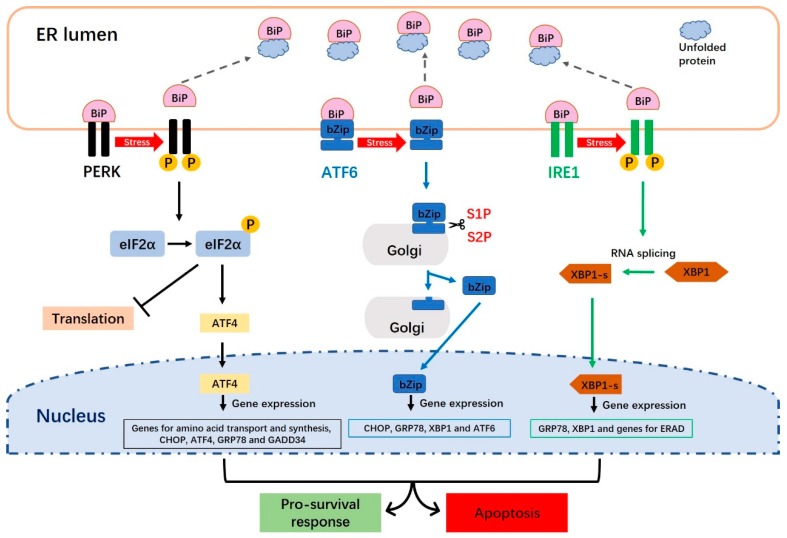
The unfolded protein response (UPR) signaling pathways. There are three distinct UPR signaling pathways in mammalian cells: The PERK, ATF6 and IRE1 pathways. Under normal conditions, the endoplasmic reticulum (ER) molecular chaperone, BiP, directly interacts with PERK, ATF6 and IRE1. Upon activation of the UPR, PERK undergoes dimerization and autophosphorylation, and then phosphorylates eIF-2α to prevent the initiation of translation and block new proteins from being produced in the cytoplasm. The PERK-phosphorylated eIF2α also activates ATF4, which translocates to the nucleus and triggers the transcription of genes required to restore ER homeostasis. When BiP is separated from ATF6, the latter factor moves to the Golgi apparatus, where it is cleaved into its active form by S1P and S2P. Cleavage of ATF6 produces a soluble basic leucine zipper (bZip) transcription factor. Active ATF6 (bZip) translocates to the nucleus and induces the transcription of ER stress-response genes via ERSE-1 and -2. The IRE1 endoribonuclease is activated through dimerization and transphosphorylation. This leads to the removal of a 26-nucleotide intron from the premature unspliced XBP1 (XBP1-u) gene form to produce the spliced XBP1 (XBP1-s) form. XBP1-s moves to the nucleus and induces UPR-responsive genes.

Three ER transmembrane proteins (PERK, ATF6 and IRE1) and the ER molecular chaperone immunoglobulin-binding protein, BiP (also known as glucose-regulated protein 78 or GRP78), trigger the UPR response to ER stress [17]. The canonical response to the accumulation of unfolded proteins in the ER involves the association (and thus sequestration) of GRP78/BiP, PERK, ATF6 and IRE1 [11]. Under normal physiological (unstressed) conditions, GRP78/BiP directly interacts with PERK, ATF6 and IRE1. However, an increase in misfolded/unfolded proteins separates GRP78/BiP from these inducers, to activate the UPR signaling pathways (Figure 2). PERK signaling decreases the translocation of new proteins into the ER lumen and prevents protein overloading, while the ATF6 and IRE1 pathways regulate the transcriptional activation of various genes, including those responsible for increasing the translocation, protein-folding, export, degradation, and other functions of the ER [19].

#### 2.2.1. The PERK Signaling Pathway

PERK is an ER transmembrane sensor protein that binds to BiP and is found in the ER lumen. Under ER stress, PERK releases BiP and triggers the dimerization and phosphorylation of eukaryotic translation initiation factor 2 alpha (eIF2α), leading to inhibition of protein synthesis (Figure 2). The PERK-mediated phosphorylation of eIF2α at ser51 initiates translation [20]. Phosphorylated eIF2α binds the guanine nucleotide exchange factor, eIF2B, to interfere with the cell’s ability to exchange guanosine diphosphate (GDP) with guanosine triphosphate (GTP) to form eIF2-GTP tRNA [18,21]. Thus, phosphorylation of eIF2α is thought to be an important step in reducing the synthesis of new proteins in the ER, releasing ER stress and restoring ER homeostasis [5,21]. PERK-phosphorylated eIF2α also activates ATF4 (activating transcription factor 4), which is key UPR-mediated gene expression. The transcriptional activity of ATF4 induces both cell survival and cell death programs. Under mild ER stress, ATF4 induces cell survival by triggering genes involved in stress responses, protein secretion and amino-acid metabolism [22]. Under prolonged stress, however, PERK signaling promotes cell apoptosis by increasing ATF4 and C/EBP homologous protein (CHOP) [1,23].

#### 2.2.2. The ATF6 Signaling Pathway

ATF6 is an ER type II transmembrane protein; it comprises an N-terminal cytoplasmic region that includes bZip and DNA transcriptional activation domains, and a C-terminal region that is exposed to the lumen of the ER [19]. There are two mammalian homologs of ATF6: ATF6-α and ATF6-β [19,24]. Under normal conditions, ATF6 maintains its resting form in the ER and is associated with BiP. When the accumulation of unfolded or misfolded proteins occurs, however, ATF6 is sequestered away from BiP and is translocated from the ER to the Golgi apparatus, where it is cleaved into its active form by site-1 protease (S1P) and site-2 protease (S2P) (Figure 2). This cleavage of ATF6 produces a soluble basic leucine zipper (bZip) transcription factor (i.e., cleaved ATF6). This active ATF6 moves to the nucleus, where it induces transcriptional activation of ER stress-response genes together with ER stress-response elements 1 and 2 (ERSE-1 and 2). The important target genes of ATF6 during the UPR include *GRP78/BiP*, *XBP1* (X-box binding protein-1) and *CHOP*. Over-expression of cleaved ATF6 has also been shown to activate the transcription of XBP1 [19], which is an important product in IRE1 signaling.

#### 2.2.3. The IRE1 Signaling Pathway

As an ER-resident transmembrane protein, IRE1 has a serine-threonine kinase domain and an endoribonuclease domain [25]. Under non-stressed conditions, IRE1 interacts with BiP and maintains an inactive form. Under ER stress, however, IRE1 is sequestered away from BiP and shifts to its active form. The endonuclease activity of IRE1, which is activated through its dimerization and transphosphorylation, removes a 26-nucleotide intron from the prematurely unspliced *XBP1* gene form (*XBP1*-*u*) to produce the spliced *XBP1*-*s* form (Figure 2). XBP1-s is usually regarded as an appropriate marker for the induction of the IRE1 arm of the UPR, because XBP1 is spliced exclusively under ER stress conditions [26]. Unlike the XBP1-u protein, which is quickly degraded, XBP1-s has a transcriptional activation domain in its C-terminus [27,28]. The XBP1-s protein translocates to the nucleus and binds to the specific promoter elements, such as the ERSE (ER stress response element) and unfolded protein response elements, to trigger transactivation of UPR target genes, such as those whose products are involved in protein folding, secretion and degradation (Figure 2).

## 3. Endoplasmic Reticulum Stress and the Unfolded Protein Response Are Intrinsic in Mammalian Oocytes and Preimplantation Embryos

### 3.1. Endoplasmic Reticulum Stress and the Unfolded Protein Response in Oocytes

XBP1 plays a critical role as a potent coordinator of ER stress and may assist in porcine oocyte maturation, early embryo development and embryonic genome activation [4]. It had been reported that the ER stress signaling pathway was essential for mouse oocyte maturation [16,29]. In mice, endogenously expressed XBP1 protein was reported to localize principally in the nucleus and weakly in the cytoplasm of germinal vesicle (GV)-stage oocytes. Assessment along the meiotic stages revealed that XBP1 localized to the spindle microtubules in M I (metaphase I)-stage oocytes, but the signal at the spindle microtubules weakened gradually in M II (metaphase II)-stage oocytes [16]. The classic ER stress marker genes, *ATF4*, *ATF6*, *XBP1*, *HSPA 5* and *GRP78*, have been detected in immature or mature oocytes of in vitro and in vivo derivations [29,30]. Porcine XBP1 protein was found to be weakly expressed in M II-stage oocytes, whereas it was localized mainly in the nucleus and weakly in the cytoplasm of GV-stage oocytes [4]. RT-PCR showed that the mRNAs for porcine *XBP1-u* and *XBP1-s* were clearly detected in GV-stage oocytes, but that only the mRNA for *XBP1-u* was detected in M I- and M II-stage oocytes [4]. This suggests that porcine XBP1 may play a significant role in oocyte maturation. In a recent study examining the expression pattern of UPR markers during porcine oocyte maturation in vitro, Park et al. [3] reported that the *BiP/GRP78* mRNA expression level was significantly increased in cumulus-oocyte complexes (COCs) at 44 h of maturation culture relative to 22 h of maturation culture. In addition, the protein expression levels of UPR signaling markers (e.g., BiP/GRP78, ATF4, p50ATF6 and CHOP) were significantly higher in COCs at 44 h than at 22 h. These results suggest that ER stress and UPR signaling are intrinsic in mammalian oocytes, and that their proper function could be essential for oocyte maturation and oocyte quality.

### 3.2. Endoplasmic Reticulum Stress and the Unfolded Protein Response in Preimplantation Embryos

It had been reported that the mechanisms responsible for controlling ER stress affect preimplantation embryo development [4]. For example, the ER stress-mediated activation of UPR signaling impairs preimplantation embryo development in vitro [31] and influences post-implantation embryo development [32,33]. In mice, knockout of UPR-associated genes, such as *GRP78/BiP* and *Ppp1r15/Gadd34*, was reported to have negative effects on embryo development, suggesting that ER chaperones and ER stress signaling play important roles in preimplantation embryos [34,35]. In mouse embryos, ER stress signaling was found at the 1-cell stage and was very abundant at the blastocyst stage [36]. XBP1 is a key transcription factor that regulates a subset of genes that are active during ER stress [16]. Zhang and colleagues reported that the XBP1 signal was higher in the nucleus than the cytoplasm at the 2-, 4-, 8-cell, morula, and blastocyst stages, as assessed by fluorescence staining. RT-PCR analysis showed that the mRNAs for *XBP1-u* and *XBP1-s* were found at the 2-cell to blastocyst stages, whereas only *XBP1-u* was detected at the 1-cell stage [16]. In an analysis of ER stress response-associated genes throughout preimplantation development, Abraham et al. reported that the mRNAs for *PERK*, *ASK1*, *ATF4*, *ATF6*, *GRP78/BiP*, *CHOP*, *GADD34* and *IRE1* were detected at all stages of mouse preimplantation development [26]. In addition, the mRNAs for *BiP*, *ATF6* and PERK pathway-related genes (e.g., *PERK*, *ATF4* and *CHOP*) were also detected in blastocyst-stage mouse embryos [37].

In pig embryos derived from parthenogenetic activation (PA) [4], the mRNAs for *XBP1-s* and *XBP1-u* were clearly detected at the 4-cell, morula and blastocyst stages, whereas only *XBP1-u* was found at the 1- and 2-cell stages. The porcine XBP1 protein was localized strongly in the nucleus and weakly in the cytoplasm during the 4-cell, morula and blastocyst stages. Moreover, the evidence suggests that ER stress-induced XBP1 splicing may regulate early embryonic genome activation in pigs [4]. Dicks et al. evaluated ER stress in both early- and late-cleaving embryos by measuring the RNA abundance of *XBP1* and *GRP78* using real time PCR and immunofluorescence technologies [38]. In buffalo in vitro fertilization (IVF)-derived embryos, the mRNAs for the ER chaperone-encoding genes, *GRP78* and *GRP94*, were detected at the blastocyst stage [14]. ER stress-associated mRNAs, including *GRP78*, *EDEM*, *ATF6*, *IRE1*, *ATF4*, *CHOP* and *XBP1*, were also reportedly expressed in bovine IVF-derived embryos [12,39].

ER stress and UPR signaling can also be detected in embryos derived from somatic cell nuclear transfer (SCNT). It appears that ER stress is stronger in electrofusion-derived SCNT embryos compared to IVF-derived embryos and Sendai virus-mediated SCNT embryos [39]. RT-PCR revealed that electrofusion- or Sendai virus-mediated SCNT embryos clearly express the mRNAs for *XBP1-u* and *XBP1-s*. The relative transcript levels of *XBP1-s* to total *XBP1* were reported to be significantly higher in electrofusion-mediated SCNT embryos than in Sendai virus-mediated SCNT embryos [39] or IVF-derived embryos [15]. The mRNA expression levels of ER stress-associated genes, such as *GRP78*, *EDEM* and *ATF6*, were markedly higher in electrofusion-mediated SCNT embryos than IVF-derived or Sendai virus-mediated SCNT embryos [39]. The expression of functional XBP1 protein was detected at all stages in porcine SCNT-derived embryos, and the mRNAs for *XBP1-u* and *XBP1-s* were detected in porcine SCNT-derived blastocysts [13]. Overall, these findings reveal that ER stress and the UPR pathways are intrinsic in IVF-, PA- and SCNT-derived embryos of many species, and thus are very likely to play important roles in preimplantation embryo development. The intrinsic ER stress and UPR signaling pathway markers that have been identified in mammalian oocytes and/or preimplantation embryos are presented in Table 1.

## 4. Activation and Induction of Endoplasmic Reticulum Stress in Oocytes and Preimplantation Embryos

Mammalian oocytes and preimplantation embryos are usually sensitive to variations in many exogenous factors, including temperature, osmotic stress, shear stress, chemical exposure, oxidative stress, etc. [11,43]. All of these factors are known to induce ER stress [17], and reduce embryo developmental potential through alterations in gene expression, epigenetic mechanisms and metabolism [44,45].

During oocyte maturation and early embryo development, various enzymes and metabolic pathways produce endogenous reactive oxygen species (ROS) [46]. The accumulation of ROS in cells leads to cell membrane lipid peroxidation, blockade of RNA transcription, damage of DNA and decreases in protein synthesis [47,48,49]. It has been reported that oxidative stress can induce ER stress and UPR signaling by impeding correct protein folding and calcium homeostasis [50]. Oxidative stress can decrease the formation rate of bovine IVF-derived blastocysts by upregulating the mRNA expression levels of *IRE1*, *ATF4*, *ATF6* and *CHOP* [12]. ER stress and oxidative stress are usually found together [43,51]; ER stress can produce ROS [52], whereas ROS can induce ER stress by disturbing correct protein folding/transport and altering calcium homeostasis [50].

Tunicamycin (TM) is a chemical reagent that inhibits N-glycosylation, which is often essential for protein folding; TM is generally used to induce ER stress [53,54]. TM has been reported to negatively affect oocyte maturation and embryo development by promoting ER protein misfolding and inducing apoptosis in mice, pig and cattle [4,13,14,16,37,40]. TM-induced ER stress usually has a negative influence on embryo development. For example, mouse embryos treated with more than 5 μg/mL TM were completely blocked at the 2-cell stage and failed to develop into blastocysts [16], while bovine and pig PA- or IVF-derived embryos failed to develop to the blastocyst stage when exposed to 5 μg/mL TM during in vitro culture [4,14,55]. In porcine SCNT-derived embryos, in contrast, as little as 1 μg/mL TM was found to block the ability of embryos to develop to the blastocyst stage [13]. This suggests that SCNT-derived embryos are more sensitive to TM than IVF-derived embryos, perhaps because cloned embryos exposed to electrofusion-mediated activation are subject to an increased ER stress response [13].

Electrofusion, which is the most common method employed to create a cloned embryo, typically increases intracellular calcium ions, decreases maturation-promoting factor activity and enhances improper nuclear remodeling [39,56]. SCNT-derived embryos derived from electrofusion reportedly undergo activation of ER stress and UPR signaling via upregulation of the mRNAs for *XBP1-s*, *GRP78* and *ATF6*, leading to decreases in the developmental competence and quality of SCNT embryos [39]. Similarly, the author of a porcine SCNT study speculated that the nuclear transfer process used during SCNT (e.g., electrofusion) could induce extra ER stress [57]. Therefore, it may be useful to decrease ER stress during SCNT embryo production by bypassing electrofusion, as a means to improve SCNT embryo development.

Osmotic stress, cryopreservation and shear stress during embryo handling can also induce the ER stress response. Culture media of an appropriate osmolality has been shown to improve oocyte maturation and embryo development [58,59], whereas hyperosmolarity of the culture media has been shown to decrease embryo developmental capacity by inducing ER stress and apoptosis [16]. For example, the addition of 50 mM sorbitol to the culture medium completely arrested mouse embryos at the 2-cell stage, while the addition of 25 mM sorbitol significantly decreased the blastocyst formation rate by increasing XBP1 protein expression [16]. Cryopreservation can also induce ER stress and UPR pathway activation. Zhao et al. reported that the protein level of XBP1 was significantly higher in vitrified-warmed mouse oocytes than in fresh mouse oocytes, suggesting that the freeze-thawing procedure triggers ER stress in mouse oocytes [40]. Similarly, sheep COCs subjected to vitrification showed increased expression levels of ER stress markers, such as the mRNAs for *ATF4*, *ATF6*, *GRP78* and *CHOP*, when compared to controls, suggesting that sheep oocyte cryopreservation is associated with ER stress and activation of the UPR signaling pathways [42]. Embryo pipetting often produces shear stress, which can damage oocytes and negatively impact embryo development [60]. Although transient shear stress may not negatively influence embryos, prolonged or repeated handling can have such effects [61]. The IRE1 arm of the UPR pathway is reportedly activated in response to embryo collection techniques [26]. Overall, the studies described above indicate that ER stress and the UPR signaling pathways are inducible and activated in mammalian oocytes and preimplantation embryos. The impacts of common ER stress activators on mammalian oocyte maturation and embryo development are summarized in Table 2.

## 5. Endoplasmic Reticulum Stress Induces Apoptosis 

### 5.1. Three Main Canonical Apoptosis Pathways Are Induced by Endoplasmic Reticulum Stress

**Figure 3:**
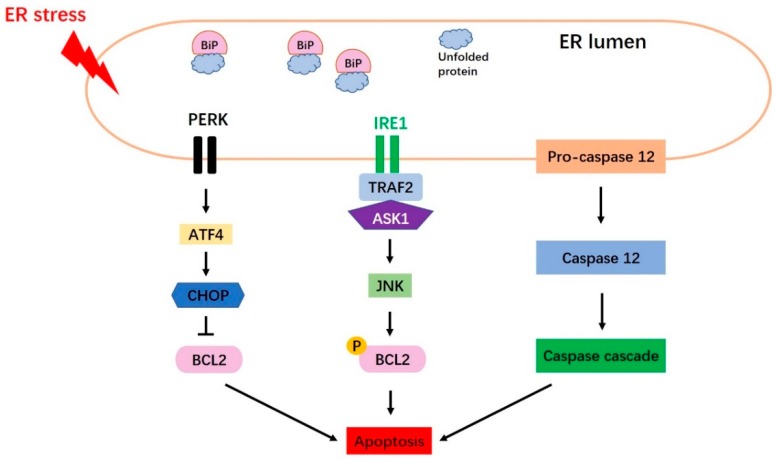
Schematic diagram of apoptosis induced by Endoplasmic Reticulum (ER) stress. Three main pathways of ER stress-mediated pro-apoptotic signaling are shown, namely the CHOP pathway, the JNK pathway and the caspase 12 pathway.

UPR signaling is extremely important for the restoration of ER homeostasis and the maintenance of normal ER function. When misfolded or unfolded proteins accumulate in the ER lumen, UPR signaling is activated to ameliorate this source of ER stress. However, if misfolded or unfolded protein accumulation is persistent and the stress cannot be relieved, apoptosis is induced by activation of the CHOP, Jun N-terminal kinase (JNK), and caspase 12 [25,62,63] pathways (Figure 3).

#### 5.1.1. The CHOP Pathway

CHOP, which is encoded by growth arrest- and DNA damage-inducible gene 153 (*GADD153*), was identified as a factor that responds to DNA damage [25]. Under conditions of severe or prolonged ER stress, all three arms of the UPR can induce CHOP transcription; however, the PERK-eIF2α-ATF4 pathway has been shown to be essential for upregulating CHOP protein expression. BiP is released from PERK to trigger ATF4, leading to the upregulation of CHOP expression (Figure 3). CHOP mainly induces apoptosis through downregulation of the anti-apoptotic gene, *BCL2* [19,25].

#### 5.1.2. The JNK Pathway

JNK is activated by the IRE1-TRAF2-ASK1 pathway (Figure 3). IRE1 binds to TRAF2 (TNF receptor-associated factor 2) and ASK1 (apoptosis signal-regulating kinase 1), leading to the activation of JNK and ASK1 [62]. ER stress-activated JNK triggers apoptosis mainly by phosphorylating ER-localized BCL2 [64].

#### 5.1.3. The Caspase 12 Pathway

Caspase 12 is an ER membrane-localized pro-apoptotic cysteine protease [19] that acts as a key mediator of ER stress-induced apoptosis [65] (Figure 3). Caspase 12 is activated by m-Calpain in the cytoplasm [66], or potentially via caspase 7-mediated cleavage [67]. After its release from the ER membrane, caspase 12 associates with caspase 9, which may lead to caspase 3 activation and subsequent apoptosis [19].

### 5.2. Endoplasmic Reticulum Stress-Mediated Apoptosis in Oocytes and Preimplantation Embryos 

Apoptosis plays important roles in mammalian oocyte maturation and preimplantation embryonic development [68]. In the latter context, apoptosis may contribute to embryonic loss [69] and affect some cellular responses [70] in embryos produced both in vitro and in vivo. ER stress-induced apoptosis has been reported in mammalian oocytes and IVM-derived embryos. The apoptotic index in blastocyst-stage mouse embryos treated with 5 μg/mL TM was found to be significantly higher than that in the TM-untreated group [16]. In addition, a higher concentration of ER stress inducer (e.g., 50 mM sorbitol) was also reported to significantly increase the apoptosis rate in mouse blastocysts compared with the control group, as assessed by the TUNEL assay [16]. Mouse oocyte cryopreservation is associated with ER stress, and the level of caspase 12 protein was reported to be significantly higher in both vitrified oocytes and vitrified oocytes treated with TM, compared to non-vitrified group [40]. In buffalo IVF-derived blastocysts, TM treatment increased the relative mRNA expression of *BAX* and the TUNEL-positive cell rate compared to the TM-untreated group [14]. Treatment with 20% O_2_ significantly increased the apoptosis rate of bovine early embryos through the activation of ROS and ER stress [12]. In porcine SCNT-derived embryos, blastocysts derived from the TM-treated group exhibited a significantly increased apoptosis rate along with upregulation of the mRNA for pro-apoptotic *BAX* and downregulation of the mRNA for anti-apoptotic *BCL2* [13]. The higher apoptotic index of SCNT-derived embryos compared to IVF-derived embryos may be attributed to the increased activation of ER stress seen in the former [15,39]. Taken together, these studies show that ER stress (e.g., those induced by TM treatment, hyperosmosis or cooling) can trigger apoptosis in oocytes and/or preimplantation embryos.

## 6. Relief of Endoplasmic Reticulum Stress Reduces Apoptosis, Improves Oocyte Maturation and Enhances the Developmental Potential of Embryos

**Figure 4:**
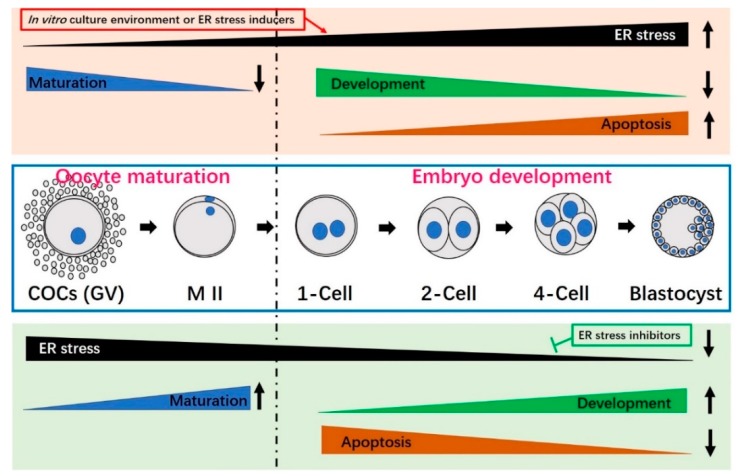
The influences of endoplasmic reticulum (ER) stress on oocyte maturation and preimplantation embryo development. Oocyte maturation and/or embryo culture environments associated with prolonged or severe ER stress (e.g., treatment with the ER stress inducer, TM) can significantly reduce oocyte maturation, decrease embryo developmental potential and increase apoptosis (top box with light pink color). When ER stress is inhibited by an ER stress inhibitor (e.g., TUDCA), maturation and development improve and the apoptotic index decreases (bottom box with light green color). Oocyte maturation is shown to the left of the broken black line, while embryo development is presented on the right. COCs, cumulus oocyte-complexes; GV, germinal vesicle; M II, metaphase II.

The ER stress inhibitor-induced decrease of ER stress-induced UPR signaling not only improves oocyte maturation and preimplantation embryo development potential, it also prevents ER stress-mediated apoptosis (Table 3, Figure 4). Tauroursodeoxycholic acid (TUDCA), which is a bile acid that acts as a potent chemical chaperone to inhibit ER stress in vitro [71], has been widely used to alleviate ER stress during in vitro oocyte maturation and/or embryo development. The beneficial role of TUDCA is typically attributed to suppression of the UPR [72,73].

TUDCA was reported to significantly improve porcine oocyte maturation by triggering the MAPK pathway and enhance the developmental capacity of early PA-derived embryos by preventing ER stress-induced apoptosis [4]. Inhibition of ER stress by supplementation with TUDCA reportedly reduces the incidence of DNA double-strand breaks (DSBs) and improves preimplantation embryo development [38]. Incubation of porcine IVF-derived embryos with TUDCA was shown to improve the blastocyst formation rate and total cell and inner cell mass (ICM) cell numbers, while decreasing the apoptosis rate and the mRNA level of pro-apoptotic *BAX* [55]. Similarly, in cattle, TUDCA supplementation of the culture medium can enhance the blastocyst formation rate, trophectoderm proportion and cell survival [12]. TUDCA (50 μM) improved the cleavage rate of buffalo IVF-derived embryos and attenuated TM-induced apoptosis by decreasing the expression levels of *BAX* and ER chaperones [14]. In mice, the addition of TUDCA to the culture medium improved the rate at which 2-cell embryos developed to blastocysts, reduced apoptosis [16] and improved the implantation and live birth rates of transferred embryos [57]. TUDCA was also shown to improve the viability and subsequent embryo developmental potential of vitrified-warmed mice oocytes by reducing cryopreservation-induced ER stress [40].

The blastocyst formation rate, total cell number, ICM cell number and apoptotic rate were significantly lower and higher in electrofusion-mediated SCNT embryos compared to Sendai virus-mediated SCNT embryos or IVF-derived embryos, respectively; this reflects the increased activation of ER stress by the electrofusion process, suggesting that ER stress plays a negative role in early SCNT-derived embryos [39]. However, TUDCA treatment significantly enhanced the formation rate and quality of electrofusion-mediated SCNT blastocysts by reducing apoptosis and increasing cell numbers [39]. The presence of TUDCA in porcine in vitro maturation (IVM) medium did not improve the cleavage or blastocyst formation rates of SCNT embryos, but inclusion of TUDCA in the in vitro culture (IVC) medium appeared to enhance the blastocyst formation rate and quality of porcine SCNT-derived embryos by alleviating ER stress, reducing the ROS level, increasing the GSH level and decreasing apoptosis [13]. A recent study in bovine SCNT-derived embryos showed that TUDCA treatment of the donor cell can also significantly enhance the fusion rate, cleavage rate, blastocyst formation rate and total cell number while decreasing the apoptotic index [41]. These studies collectively suggest that ER stress is a common event in mammalian oocyte IVM and/or embryo IVC systems, and that relieving ER stress by TUDCA treatment can improve oocyte maturation, enhance embryo developmental potential and reduce apoptosis.

Melatonin (N-acetyl-5methoxytryptamine), which is an indole that is mainly synthesized from tryptophan by the pineal gland in animals, contributes to many important physiological functions, such as sleep, temperature regulation, metabolism, the circadian rhythm and seasonal reproduction [74,75,76,77,78]. It is also a free radical scavenger, anti-oxidant and anti-apoptotic factor [79]. Unlike other free radical scavengers, melatonin is multifunctional and universal: It can directly obliterate toxic oxygen derivatives, prevent ROS generation, and induce anti-oxidative enzymes [80,81,82]. Melatonin reportedly has beneficial influences when added to the culture medium during the oocyte maturation and embryo development of pigs, cattle and mice [80,83,84]. As mentioned above, oxidative stress (e.g., ROS) contributes to UPR activation; thus, melatonin can exert its functions on cell viability by regulating the UPR signaling pathways or reducing ER stress via its anti-oxidant and anti-apoptotic properties. Although melatonin has repeatedly been used to reduce ER stress in cells [85,86], relatively few studies have focused on the mechanisms through which melatonin decreases ER stress during mammalian oocyte maturation and/or preimplantation embryo development. One study in a porcine oocyte maturation system revealed that adding melatonin to the maturation medium improved meiotic maturation by reducing ER stress, suggesting that melatonin critically modulates UPR signaling and reduces ER stress during oocyte IVM in pig [3]. Numerous factors involved in in vitro culture can induce ER stress, which damages oocyte maturation and embryo development. The use of melatonin as an anti-oxidant and anti-apoptotic factor could relieve ER stress by modulating UPR signaling pathways, which could yield improved oocyte maturation and/or embryo developmental potential. The mechanisms through which melatonin acts on ER stress and the UPR signaling pathways during mammalian oocyte maturation and preimplantation embryo development await further clarification.

In addition to TUDCA and melatonin, the HDAC (histone deacetylase) inhibitor, valproic acid, or glutathione (GSH) can also reduce ER stress by regulating the UPR pathways. In bovine SCNT embryos, valproic acid was reported to significantly reduce the mRNA levels of *XBP1-s* (an ER stress marker), *CHOP* and *BAX* (a pro-apoptotic gene) while increasing those of the ER molecular chaperone, *BiP*, and the anti-apoptotic gene, *BCL-xl*; these findings suggested that valproic acid could improve the developmental potential of bovine SCNT-derived embryos by alleviating ER stress and reducing apoptosis [15]. GSH reportedly inhibits ER stress during mouse embryo development [37]. It was also found to significantly decrease the mRNA expression levels of *ATF6*, *ATF4*, *BiP*, *CHOP* and *PERK*, and increase the cell numbers and apoptosis index in blastocysts; thus, GSH appears to improve the developmental capacity and quality of mouse embryos by alleviating ER stress and apoptosis [37]. Salubrinal, a selective eIF2α dephosphorylation inhibitor, has been reported to protect cells from ER stress [87]. In mouse COCs, palmitic acid-mediated ER stress reportedly impairs mitochondrial membrane potential, pentraxin-3 secretion and embryonic development; however, salubrinal treatment was shown to significantly improve these deficiencies by reducing *ATF4*, *ATF6* and *XBP1* gene expression levels, suggesting that salubrinal could reverse the cellular dysfunctions induced by ER stress and improve oocyte development [29].

The studies above show that supplementation of the in vitro culture medium with chemical inhibitors of ER stress could serve as a beneficial approach to preventing ER stress-induced oocyte damage and alterations of embryo development. The effects of various ER stress inhibitors on mammalian oocyte maturation and embryo development are summarized in Table 3. ER stress often occurs alongside other stress responses, particularly oxidative stress [11]. ER stress itself induces ROS generation, whereas ROS contributes to the activation of ER stress and UPR signaling, so it is possible that some anti-oxidants may hold promise as potential inhibitors of ER stress.

## 7. Conclusions

Oocyte IVM and embryo IVC systems are essential for the successful production of live animals via in vitro-produced (IVP) embryos. In vitro culture conditions can increase numerous stresses that have the ability to damage the oocyte maturation and preimplantation embryo development of IVP embryos. Mammalian oocytes and embryos are highly sensitive to these diverse exogenous stresses. Many factors can negatively impact the ER, new protein synthesis and protein processing, initiating ER stress and the UPR signaling responses. As an adaptive response to ER stress, the UPR can facilitate clearance of the unfolded or misfolded proteins and cell survival. However, under prolonged stress or failure of the UPR, apoptosis is induced. ER stress usually plays a negative role in oocyte maturation and/or early embryo development (Table 2, Figure 4). Fortunately, oocyte quality and embryonic developmental potential may be improved by the addition of ER stress-reducing chemicals to the in vitro culture medium (Table 3, Figure 4). The ER stress inhibitor, TUDCA, has been widely used to attenuate ER stress during mammalian oocyte maturation and embryo development. In the future, we need to search for more ER stress inhibitors that can improve oocyte and embryo quality and continue studying the mechanisms through which ER stress and UPR signaling affect mammalian oocyte maturation and preimplantation embryo development.

In addition, although success rate of human IVF and intracytoplasmic sperm injection (ICSI) have improved progressively, the efficiency of assisted reproductive technology (ARTs), based on practical embryo production, is still low [88]. Embryos must be manipulated in vitro during human ARTs, and adverse in vitro condition mentioned above in mammalian embryo development in vitro could induce ER stress and UPR signaling pathways that may negatively impact human embryo development. Thus, understanding the mechanistic relationships between ER stress and in vitro development during human embryo production in vitro could help the improvement of human ARTs. 

## Figures and Tables

**Table 1:** Intrinsic endoplasmic reticulum stress markers identified in mammalian oocytes and preimplantation embryos.

Species	Stage (Derivation)	ER Stress Markers		Refs
		Genes	Proteins	
Mouse	Oocytes	*ATF4*, *ATF6* and *BiP/GRP78*		[30]
Mouse	Oocytes	*XBP1*, *ATF4*, *ATF6* and *HSPA5*		[29]
Mouse	Oocytes (vitrified)		XBP1	[40]
Mouse	Oocytes/blastocysts	*XBP1-u* and *XBP1-s*	XBP1	[16]
Mouse	Blastocysts	*ATF4*, *ATF6*, *BiP*, *CHOP* and *PERK*		[37]
Mouse	Blastocysts	*XBP1*, *BIP*, *IRE1*, *ASK1*, *ATF4*, *ATF6*, *CHOP* and *PERK*		[26]
Mouse	Blastocysts	*XBP1*	BiP	[31]
Pig	Oocytes	*BiP*, *ATF4*, *XBP1-u*, *XBP1-s* and *CHOP*	ATF4, ATF6, BiP/GRP78, P90ATF6, P50ATF6 and CHOP	[3]
Pig	Oocytes and 1-cell to blastocyst-stage embryos (PA)	*XBP1-u* and *XBP1-s*	XBP1	[4]
Pig	Embryos (day 5)/blastocysts (PA)	*XBP1* and *GRP78*		[38]
Pig	Blastocysts (SCNT)	*XBP1-u* and *XBP1-s*	XBP1	[13]
Bovine	Blastocysts (IVF)	*XBP1-u*, *XBP1-s*, *IRE1*, *ATF4*, *ATF6* and *CHOP*		[12]
Bovine	Blastocysts (IVF and SCNT)	*XBP1-u*, *XBP1-s* and *CHOP*		[15]
Bovine	Blastocysts (IVF and SCNT)	*XBP1*, *BiP/GRP78*, *EDEM*, *ATF4*, *ATF6*, *CHOP* and *IRE1*		[39]
Bovine	Blastocysts (SCNT)	*BiP*, *CHOP* and *IRE1*		[41]
Buffalo	Blastocysts (IVF)	*GRP78* and *GRP94*		[14]
Sheep	Oocytes	*ATF4*, *ATF6*, *GRP78* and *CHOP*		[42]

Refs: References.

**Table 2:** The impact of endoplasmic reticulum stress inducers on mammalian oocyte maturation and embryo development.

Inducer	Treatment	Species	Results	Refs
TM	0.1–1 μM during IVC	Pig	Reduces porcine SCNT-derived blastocyst formation rate, decreases total cell and ICM cell numbers, increases *XBP1-s* mRNA expression and apoptosis	[13]
TM	0.5 μM during IVC	Mouse	Reduces blastocyst formation rate, increases ROS and apoptosis	[37]
TM	0.5 μM during IVC	Mouse	Prevents blastocyst formation, increases the levels of cleaved caspase 3 protein and the mRNA for *XBP1-s*	[31]
TM	1 μM in freezing medium	Mouse	Increases XBP1 and caspase 12 protein expression levels	[40]
TM	1–2 μM during IVC	Buffalo	Decreases blastocyst formation rate and cell numbers, increases apoptosis	[14]
TM	2 μM during IVC	Pig	Induces active XBP1 in nuclei of 4-cell stage PA-derived embryos, reduces cleavage rate, blastocyst formation rate, and cell numbers in blastocysts	[4]
TM	1–5 μM during IVC	Pig	Reduces porcine IVF-derived embryo development by reducing the rates of cleavage and blastocyst formation	[55]
TM	1–5 μM during IVM	Pig	Reduces oocyte maturation in pig by increasing the expression levels of BiP, ATF4 and ATF6	[3]
TM	1–10 μM during IVC	Mouse	Reduces blastocyst formation rate, increases apoptotic index	[16]
Sorbitol	10–75 mM during IVC	Mouse	Reduces blastocyst formation rate, increases apoptotic index	[16]
Oxidative stress	Embryos cultured under 20% O_2_	Bovine	Reduces bovine IVF-derived blastocyst formation rate; increases *XBP1-s*, *ATF4*, *ATF6*, *CHOP* and *IRE1* gene expression levels and the apoptotic index	[12]
Shear stress	Embryo collection	Mouse	Triggers transient activation of the XBP1 arm of ER stress	[26]
Electro-fusion	SCNT embryos produced by electrofusion	Bovine	Reduces SCNT-derived embryonic development by increasing *XBP1-s*, *CHOP*, *GRP78* and *EDEM* gene expression levels and the apoptotic index, while reducing the blastocyst formation rate and the total and ICM cell numbers	[39]

Refs: References.

**Table 3:** The positive influence of Endoplasmic Reticulum stress inhibitor supplementation during IVM/IVC on mammalian oocyte maturation and/or embryo development.

Inhibitor	Treatment *	Species	Results	Refs
TUDCA	50 μM during IVC	Mouse	Improves embryo development and increases the implantation and live birth rates of transferred mouse embryos	[57]
TUDCA	50 μM during IVC	Mouse	Improves the blastocyst formation rate and reduces apoptosis	[16]
TUDCA	50 μM in the freezing medium	Mouse	Enhances the viability and embryonic developmental capacity of vitrified-warmed mouse oocytes by reducing ER stress	[40]
TUDCA	50 μM during IVC	Bovine	Reduces ER stress and ROS levels; improves bovine embryo development	[12]
TUDCA	50 μM during IVC	Bovine	Improves bovine SCNT embryo development by increasing cell numbers and reducing ER stress and apoptosis	[39]
TUDCA	50 μM during IVC	Buffalo	Attenuates apoptosis and ER stress in buffalo IVF-derived embryos	[14]
TUDCA	50 μM during IVM, 50 μM during IVC	Pig	Enhances porcine oocyte maturation and PA-derived embryo developmental potential by preventing ER stress	[4]
TUDCA	50 μM during IVC	Pig	Enhances DNA damage repair and improves porcine preimplantation embryo development by reducing ER stress	[38]
TUDCA	100 μM during IVC	Pig	Improves porcine SCNT embryonic development by attenuating ER stress and reducing apoptosis	[57]
TUDCA	100 μM (treatment of donor cells)	bovine	Improves the development of bovine SCNT-derived embryos by reducing ER stress	[41]
TUDCA	200 μM during IVC	Pig	Improves the development of porcine IVF-derived embryos by modulating ER stress-induced apoptosis	[55]
TUDCA	200 μM during IVM	Pig	Improves the quality and maturation of porcine oocytes	[3]
Melatonin	0.1 μM during IVM	Pig	Improves cumulus cell expansion and oocyte maturation by combating ER stress	[3]
Valproic acid	3 mM during IVC	Bovine	Improves the development of bovine SCNT-derived embryos by reducing ER stress and apoptosis	[15]
GSH	1 mM during IVC	Mouse	Increases the mouse blastocyst formation rate and alleviates ER stress	[37]
GSH	1 mM during IVC	Bovine	Reduces ROS levels and increases the blastocyst formation rate	[12]
Salubrinal	100 nM during IVM	Mouse	Improves pentraxin-3 secretion, mitochondrial membrane potential, and embryonic development by reducing ER stress	[29]

Refs: References. * The optimal concentrations of the ER stress inhibitors are listed.

## Abbreviations

ASK1	Apoptosis signal-regulating kinase 1
ATF4	Activating transcription factor 4
ATF6	Activating transcription factor 6
BiP	Immunoglobulin-binding protein
bZip	Basic leucine zipper
CHOP	C/EBP homologous protein
COCs	Cumulus-oocyte complexes
DNA	Deoxyribonucleic acid
eIF2α	Eukaryotic translation initiation factor 2 alpha
ER	Endoplasmic reticulum
ERAD	ER-associated degradation
ERQC	Endoplasmic reticulum quality control
ERSE	ER stress response element
GADD153	Growth arrest- and DNA damage-inducible gene 153
GDP	Guanosine diphosphate
GRP78	Glucose-regulated protein 78
GSH	Glutathione
GTP	Guanosine triphosphate
GV	Germinal vesicle
ICM	Inner cell mass
IRE1	Inositol-requiring enzyme 1
IVC	In vitro culture
IVF	In vitro fertilization
IVM	In vitro maturation
IVP	In vitro-produced
JNK	Jun N-terminal kinase
M I	Metaphase I
M II	Metaphase II
mRNA	Messenger ribonucleic acid
PA	Parthenogenetic activation
PERK	Double-stranded RNA-activated protein kinase-like ER kinase
ROS	Reactive oxygen species
S1P	Site-1 protease
S2P	Site-2 protease
SCNT	Somatic cell nuclear transfer
TM	Tunicamycin
TRAF2	TNF receptor-associated factor 2
TUDCA	Tauroursodeoxycholic acid
UPR	Unfolded protein response
XBP1	X-box binding protein-1
XBP1-s	Spliced XBP1
XBP1-u	Unspliced XBP1
